# Effects of compartment and severity of pelvic organ prolapse on voiding difficulty and overactive bladder: A case-control study with multiple outcomes

**DOI:** 10.1371/journal.pone.0348221

**Published:** 2026-05-21

**Authors:** Apisith Saraluck, Orawee Chinthakanan, Komkrit Aimjirakul, Rujira Wattanayingcharoenchai, Jittima Manonai

**Affiliations:** Division of Female Pelvic Medicine & Reconstructive Surgery, Department of Obstetrics and Gynaecology, Faculty of Medicine Ramathibodi Hospital, Mahidol University, Bangkok, Thailand‌‌; University College London, UNITED KINGDOM OF GREAT BRITAIN AND NORTHERN IRELAND

## Abstract

**Background:**

The current understanding of the relationship between different degrees of prolapse and the impact of prolapse in different compartments on voiding difficulty (VD) and overactive bladder (OAB) remains insufficient and requires further research. This study aimed to investigate the association between pelvic organ prolapse (POP) and the risk of VD and OAB.

**Methods:**

A retrospective case-control study was conducted among women diagnosed with POP at urogynaecology clinic in a university hospital from January 2018 to December 2020. Patients were divided into two case events (VD and OAB) and a control group (POP without lower urinary tract symptoms [LUTS]). Associations between the site and severity of prolapse in the case and control groups were investigated to identify the factors involved in VD and OAB.

**Results:**

The study included 151 cases of women with POP experiencing VD, 139 instances of OAB, and 151 women without LUTS in the control group. Most women with POP were postmenopausal, multiparous, and overweight. A multivariate logistic regression analysis revealed that the only factor associated with VD and OAB among women with POP was an advanced stage of anterior compartment prolapse (odds ratio [OR] 4.91, 95% confidence interval [CI] 2.36–10.25, p < 0.001, OR 2.28, 95% CI 1.09–4.78, p < 0.03, respectively).

**Conclusions:**

An advanced stage of anterior compartment prolapse was the main factor that affected VD and OAB in women with POP.

## Introduction

Pelvic organ prolapse (POP) is a pelvic floor disorder associated with various symptoms of pelvic floor dysfunction, including urinary symptoms (e.g., urinary incontinence and voiding dysfunction), defecatory symptoms, and sexual dysfunction [1]. POP involves structural changes affecting different anatomical compartments of the pelvic floor [2]. Anterior vaginal wall prolapse has been linked to lower urinary tract symptoms (LUTS), such as urinary incontinence, increased urinary frequency, or difficulties emptying the bladder [3,4]. However, several theories suggest that pelvic floor symptoms, particularly LUTS, may also be influenced by the overall support for all pelvic organs within Delancey’s three levels of pelvic support, which represent a complex and integrated support system [5]. Previous studies have generated controversy by suggesting that women with compartment-specific prolapse experience distinct LUTS patterns, although these associations are not consistently observed [6]. Two of the most debated urinary symptoms in relation to POP are overactive bladder (OAB) and voiding difficulty (VD) [7–9]. Voiding difficulty (VD) is a subjective symptom characterized by sensations of incomplete emptying, hesitancy, weak urinary stream, or straining during urination. Diagnostic evaluation is often strengthened by combining patient-reported symptoms with objective assessments, such as measurement of post-void residual urine volume [10]. Several mechanisms have been proposed to explain the relationship between POP and voiding dysfunction. Advanced anterior vaginal wall prolapse may lead to urethral kinking or functional bladder outlet obstruction, which may result in incomplete bladder emptying and voiding difficulty. In addition, some patients with significant prolapse adopt compensatory behaviors to facilitate bladder emptying, such as abdominal straining or manual reduction of the prolapse during voiding [11]. Overactive bladder (OAB), in contrast, is primarily defined by patient-reported symptoms rather than objective clinical findings.[12] OAB symptoms include urinary urgency, usually accompanied by frequency and nocturia, with or without urgency urinary incontinence. Because OAB is often considered idiopathic, the mechanisms linking OAB symptoms to pelvic floor disorders remain incompletely understood. Some hypotheses suggest that anatomical changes in pelvic support structures, such as weakening of the pelvic fascia due to ageing, childbirth, or connective tissue disorders, may contribute to bladder dysfunction. In addition, chronic mechanical distortion of the bladder or bladder neck associated with prolapse may contribute to detrusor overactivity and urgency symptoms [13,14].

In 2023, the International Urogynecological Association published the International Urogynecology Consultation Chapter 2 Committee 3 report [15], which discusses the clinical evaluation of POP and associated pelvic floor dysfunction. The report highlights that the current understanding of the relationship between prolapse severity, compartment involvement, and urinary symptom patterns remains limited and requires further research. Therefore, the present study aimed to evaluate the effects of compartment involvement and severity of POP on two common urinary outcomes, voiding difficulty and overactive bladder, in women with POP, compared with POP patients without urinary symptoms.

## Materials and methods

This retrospective chart review study included three groups of women with pelvic organ prolapse (POP): women with voiding difficulty (VD), women with overactive bladder (OAB), and women with POP without lower urinary tract symptoms (control group). Each symptomatic group (VD and OAB) was analysed separately in comparison with the same control group. This research was conducted after the approval by the Ethical Clearance Committee on Human Rights related to Researches involving Human Subjects, Faculty of Medicine, Ramathibodi Hospital, Mahidol University, approval date 12/03/2024 (MURA2024/184). Data were accessed from medical records starting on 01/04/2024, after approval from the Ethics Committee. Data from the medical records of women attending the clinic from 01/01.2018 to 31/12/2020 were reviewed. VD (Case Event 1) in this study was defined as being unable to empty the bladder, a feeling of incomplete bladder emptying, or postvoid residual urine greater than 100 mL [16]. The post-void residual (PVR) volume was measured using bladder ultrasound, performed immediately after voiding to ensure accuracy. The PVR volume was calculated based on bladder dimensions obtained through the scan, using the formula: PVR Volume = 0.52 × Height×Width×Depth [17]. This method provides a reliable and non-invasive assessment of urinary retention. Moreover, patients in this group had reported urinary difficulties at their initial visit and rated the bothersomeness score and Pelvic Floor Symptom Bother Questionnaires [18,19] (PFBQ) item 5 (“voiding difficulty”) as severe (scores 3 and 4, respectively). In this study, the term “voiding difficulty” was used to describe symptom-based difficulty in bladder emptying rather than a specific urodynamic diagnosis. OAB (Case Event 2) was defined as having urinary urgency, usually with urinary frequency and nocturia, with or without urgency urinary incontinence. Nocturia was not used as an isolated diagnostic criterion for OAB but was considered together with other storage symptoms, particularly urinary urgency. Patients in this group had reported OAB symptoms at their initial visit, mentioning frequency of urination and urgency, and rated the bothersomeness score and PFBQ items 2, 3, and 4 as severe (scores 3 and 4, respectively). Patients routinely completed pelvic floor symptom questionnaires as part of the standard clinical evaluation according to IUGA recommendations. In this study, the documentation of the patients’ symptoms and diagnoses in these events must have strictly adhered to the complete definition. The exclusion criteria were having more than one LUTS, diagnosis with urinary tract infections, unclear evidence of urinalysis, known neurological conditions affecting bladder function, and incomplete medical record data. The control group consisted of women with POP without LUTS. Patients in this group had not reported any LUTS at the initial visit and reported “No” in response to PFBQ items 2, 3, 4, and 5. Cases and controls were selected at a ratio of 1:1:1 for VD, OAB, and control groups. For the review of medical records, a patient’s baseline characteristic data, history, and important or relevant physical examinations. The degree of POP was classified according to the POP-Q system and further grouped into early stage (stage I–II) when the most distal portion of the prolapse does not extend beyond the hymen and advanced stage (stage III–IV) when the prolapse extends beyond the hymen [20]. Data were obtained through an electronic medical record review and recorded in the data collection system of female pelvic medicine and reconstructive surgery division. All eligible cases within the study timeframe were included. In the univariate analysis, comparisons were conducted using appropriate statistical tests based on the nature of the variables. Continuous variables were analyzed using independent t-tests or Mann–Whitney U tests, depending on normality. For categorical variables, chi-square tests or Fisher’s exact tests were applied as appropriate. Dichotomous variables were assessed using logistic regression to estimate odds ratios (ORs) and 95% confidence intervals (CIs). In the multivariate analysis, variables included in the logistic regression model were selected based on specific criteria: statistical significance in univariate analysis (p < 0.10) with clinical relevance as supported by previous literature. Univariate analysis was performed on the variables, followed by multivariable logistic regression to examine the association between the degree of POP and compartmental prolapse, comparing cases and controls. Missing data points were excluded from the analysis. Statistical analysis was performed using STATA version 17 [21].

The sample size was calculated using the sample size for case-control formula [22] with reference to a previous study [23] with a 1:1 ratio for cases to controls, a significance level of 0.05, a power (1-β) of 80%, and an expected effect size of odd ratio 1.9. After including possible missing data from a retrospective study, the sample size in each group was 125 cases.

## Results

The study population consisted of women with pelvic organ prolapse (POP), including 151 cases with voiding difficulty (VD), 139 with overactive bladder (OAB), and 151 with POP without lower urinary tract symptoms who served as the control group. The baseline characteristics of the patients in this study are shown in Table 1. Most participants were postmenopausal and multiparous and had a history of vaginal delivery. There was no statistically significant difference in the baseline characteristics of the cases and controls (Table 1).

**Table 1:** Baseline characteristics of patients with pelvic organ prolapse: comparison between patients with voiding difficulty (VD), overactive bladder (OAB), and controls without urinary symptoms.

	Voiding difficulty (n = 151)	OAB (n = 139)	Controls (n = 151)
age (years)	67.03 ± 8.46	65.63 ± 9.34	64.60 ± 10.26
BMI (kg/m2)	25.37 ± 3.35	25.37 ± 3.54	24.93 ± 3.19
Parity, n (%)			
Nulliparity	5 (3.31)	8 (5.76)	13 (8.61)
Parity	146 (96.69)	131 (94.24)	138 (91.39)
History of vaginal delivery, n (%)			
yes	146 (96.69)	131 (94.24)	137 (90.73)
no	5 (3.31)	8 (5.76)	14 (9.27)
Menopausal status, n (%)			
pre-menopause	5 (3.31)	6 (4.32)	9 (5.96)
post-menopause	146 (96.69)	133 (95.68)	142 (94.04)
Previous hysterectomy, n (%)			
yes	19 (12.58)	16 (11.51)	11 (7.28)
no	132 (87.42)	123 (88.49)	140 (92.72)
Previous pelvic floor repair, n (%)			
yes	2 (1.32)	4 (2.88)	2 (1.32)
no	149 (98.68)	135 (97.12)	149 (98.68)
			

The univariate analysis of anatomical variables revealed that advanced anterior compartment prolapse, advanced posterior compartment prolapse, advanced apical compartment prolapse, higher genital hiatus length, and multiple compartment prolapse were significant factors in VD, and advanced anterior compartment prolapse, higher genital hiatus length, and shorter perineal body length were significant factors in OAB (Table 2).

**Table 2:** Variables between voiding difficulty cases, OAB case and control in women with POP.

	Voiding Difficulty	Controls	p value	OAB	Controls	p value
Anterior compartment prolapse			< 0.001			0.01
early stage	47 (31.13)	103 (68.21)		75 (53.96)	103 (68.21)	
advanced stage	104 (68.87)	48 (31.79)		64 (46.04)	48 (31.79)	
Posterior compartment prolapse			0.002			0.12
early stage	105 (69.54)	128 (84.77)		108 (77.70)	128 (84.77)	
advanced stage	46 (30.46)	23 (15.23)		31 (22.30)	23 (15.23)	
Apical compartment prolapse			< 0.001			0.61
early stage	50 (33.11)	86 (56.95)		75 (53.96)	86 (56.95)	
advanced stage	101 (66.89)	65 (43.05)		64 (46.04)	65 (43.05)	
Genital hiatus length (cm.), mean sd	4.40 (1.04)	3.88 (0.87)	< 0.001	4.20 (1.10)	3.88 (0.87)	0.01
Perineal body lenghth (cm.), mean sd	2.39 (0.72)	2.63 (0.78)	0.99	2.44 (0.70)	2.63 (0.78)	0.03
Number of compartment prolapse			< 0.001			0.33
single	33 (21.85)	65 (43.05)		52 (37.41)	65 (43.05)	
multiple	118 (78.15)	85 (56.95)		87 (62.59)	85 (56.95)	

**Fig 1:**
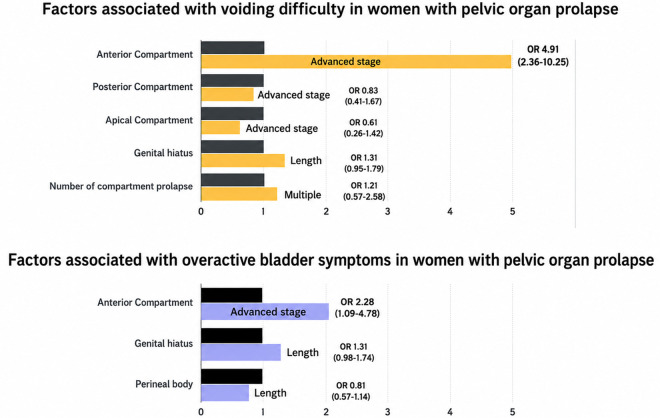
Comprehensive Analysis of Factors Associated with Voiding Difficulty and Overactive Bladder Symptoms in Women with Pelvic Organ Prolapse‌‌.

The multivariate logistic regression analysis of anatomical variables revealed that advanced-stage anterior compartment prolapse had the highest odds ratio and was the only statistically significant factor in the final model for both VD and OAB (Table 3). Specifically, advanced-stage anterior compartment prolapse is significantly associated with VD (OR 4.91, 95% CI: 2.36–10.25) and OAB symptoms (OR 2.28, 95% CI: 1.09–4.78). Other factors, including genital hiatus length, perineal body length, and multiple compartment prolapse, showed weaker or non-significant associations (Fig 1).

**Table 3:** Multivariate logistic regression analysis factors associated with voiding difficulty and OAB.

	Voiding difficulty				OAB	
	OR	95% CI	p value^a^	OR	95% CI	p value^b^
Advanced stage anterior compartment prolapse	4.91	2.36, 10.25	<0.001	2.28	1.09, 4.78	0.03
Advanced stage posterior compartment prolapse	0.83	0.41, 1.67	0.59	N/A	N/A	N/A
Advanced stage apical compartment prolapse	0.61	0.26, 1.42	0.25	N/A	N/A	N/A
Genital hiatus length	1.31	0.95, 1.79	0.09	1.3	0.98, 1.74	0.07
Perineal body lenghth	N/A	N/A	N/A	0.81	0.57, 1.14	0.22
Multiple compartment prolapse	1.21	0.57, 2.58	0.62	N/A	N/A	N/A

*Each one is adjusted by age, parity, vaginal delivery, and clinically relevant exposures.

**Reference level of each exposure: early stage of the compartment, single compartment prolapses.

^a^p value from statistical analysis comparing control group and voiding difficulty group.

^b^p value from statistical analysis comparing control group and OAB group.

## Discussion

This study investigated the association between POP characteristics and LUTS, specifically VD and OAB. The most striking finding was the strong association between advanced stage anterior compartment prolapse and VD and OAB, implying that anterior compartment prolapse might affect the voiding and storage phases of urination. The demographic characteristics of this study, with most participants being postmenopausal and having a history of vaginal delivery, are consistent with the established risk factors for pelvic organ prolapse reported in previous studies.

This finding aligns with the accepted concept that urinary tract functions are greatly affected by the anterior compartment, which involves the bladder. Furthermore, the prolapse of the anterior wall compartment exerts pressure directly on the urethra through a buttressing effect. The degree of anterior vaginal wall descent was demonstrated to obstruct urinary flow, resulting in VD. This study showed that women with advanced prolapse in the anterior compartment are nearly five times more likely to experience VD than those in the early stage of prolapse. This result aligns with a report from the US and Korea, which showed a significant positive association between advanced-stage anterior compartment prolapse and the lower urinary tract symptom tool obstructive severity score and VD symptoms [24]. Moreover, a previous investigation conducted at a high-volume urogynaecology centre reported that VD affected 25–29% of women with POP. Nevertheless, the precise correlation between LUTS and pelvic organ descent was ambiguous, and patients’ symptoms and definitions of VD were not reported [25,26]. In contrast, a study from India reported that the advanced stage of posterior compartment prolapse had a significant correlation with VD; however, it was a cohort study and did not compare cases with women with POP and normal lower urinary tract function [27]. Although the advanced stage of anterior compartment prolapse may have an impact on VD, it is critical to evaluate this specific condition in women with POP prior to treatment, particularly to exclude neurologically associated conditions such as bladder underactivity.

POP and OAB symptoms frequently coexist; however, there is inconsistent evidence regarding a correlation between the severity of the symptoms and a specific anatomical defect [28]. Most previous studies have not reported an association between the severity of anterior compartment and OAB symptoms. OAB appears to be more prevalent in stages I and II of prolapse as opposed to more advanced stages of POP, given that severe POP can induce obstructive urinary symptoms and VD [24,29]. In contrast, another study reported that a greater proportion of women with POP stage III or IV had OAB than those with POP stage I or II, which correlates with our findings [30]. Moreover, previous studies have reported that overactive bladder symptoms may improve following surgical correction of anterior compartment prolapse. Improvement rates of approximately 40–50% have been described after prolapse repair, suggesting that anatomical restoration may influence bladder function [31]. These findings support the concept that prolapse-related anatomical changes may contribute to urinary symptoms in some patients.

To the best of our knowledge, this is the first case-control study of how the severity of pelvic organ prolapse impacts VD and OAB in women with POP, a condition that is still poorly understood. This research contributes to the development of knowledge in numerous significant ways, despite the fact that the correlation between anterior compartment prolapse, VD, and OAB is consistent with existing theories. This retrospective case–control study, which involved the examination of multiple occurrences or types of events within the same population, was ideal for providing more information and elucidating the mechanisms involved in VD and OAB. This study design is particularly useful when conditions share common risk factors or exposures, because it establishes relationships between different conditions and exposures, thereby providing a more comprehensive understanding of how these interact from the same dataset. This can enhance the reliability of the findings and result in more generalisable results. The inclusion of data from a referral centre enhanced the generalisability of the results. Moreover, this study offers a quantitative assessment of the impact of anterior compartment prolapse severity on VD and OAB by providing precise odds ratios and confidence intervals. This enhances the quality of clinical decision-making in the context of risk stratification. Additional strengths of the study include a highly reliable physical examination and stage of POP in our setting, which were confirmed by experts in the field, as well as the use of a validated questionnaire and strict inclusion criteria when enrolling participants in the study. The necessity of early identification and customised management strategies, particularly prior to the consideration of surgical or non-surgical interventions, is substantiated by the results of this study. This study not only strengthens the current comprehension of the role of anterior prolapse in VD and OAB but also provides stronger clinical evidence, refined risk estimation, and methodological advancements that enhance the field’s current knowledge by integrating these elements. Our findings may also have implications for clinical counseling. Understanding the relationship between prolapse compartment involvement and urinary symptoms may help clinicians better explain symptom mechanisms to patients during preoperative assessment. This information may also contribute to addressing the knowledge gaps highlighted in recent IUGA consensus reports regarding the relationship between prolapse severity and lower urinary tract symptoms. This study has several limitations that should be acknowledged. The cross-sectional design prevents causal inferences between anterior prolapse and VD and OAB, limiting the ability to establish a direct cause-and-effect relationship. The exclusion of patients with incomplete records may have introduced selection bias. Another limitation of this study is the lack of routine urodynamic evaluation for all patients. Therefore, the underlying pathophysiological mechanisms of urinary symptoms could not be fully characterised using objective urodynamic parameters, such as bladder outlet obstruction related to POP or detrusor underactivity. Future studies with prospective, longitudinal designs, diverse populations, comprehensive confounder adjustments, and objective outcome measures are necessary to validate these findings and further explore the associations observed in this study.

## Conclusion

OAB and VD symptoms are prevalent among women with POP and are closely associated with advanced anterior compartment prolapse. The treatment of POP should be preceded by the evaluation of advanced anterior compartment prolapse and suitable comprehensive care.

## Supporting information

S1 File De-identified dataset used for statistical analysis of pelvic organ prolapse, voiding difficulty, and overactive bladder outcomes. (XLSX)
